# μ_3_-Methoxido-κ^3^ *O*:*O*:*O*-tris­(μ-l-*p*-tyrosinato-κ^3^ *N*,*O*:*O*)tris­(l-*p*-tyrosinato-κ^2^ *N*,*O*)trinickel(II,III) methanol tetra­solvate

**DOI:** 10.1107/S1600536813010696

**Published:** 2013-04-24

**Authors:** Weerinradah Tapala, Timothy J. Prior, Apinpus Rujiwatra

**Affiliations:** a Department of Chemistry, Faculty of Science, Chiang Mai University, Chiang Mai 50200, Thailand; b Department of Chemistry, University of Hull, Cottingham Road, Hull HU6 7RX, England

## Abstract

A trinuclear nickel complex, [Ni_3_(C_9_H_10_NO_3_)_6_(CH_3_O)]·4CH_4_O, was synthesized and characterized as a neutral cluster containing the incomplete cubane {Ni_3_(μ_1_-O)(μ_2_-O)_2_(μ_3_-O)} core of 2M3–1 topology. The three nickel cations show similar octa­hedral coordination, {Ni(μ_1_-O)(μ_2_-O)_2_(μ_3_-O)(μ_1_-N)_2_}; the positive charge is balanced by six tyrosinate ligands and one methoxide ion. The mean oxidation state of each Ni^II^ ion is therefore +2.33. The common coordination modes, chelating (*via* the amino N and the carboxyl­ate O atoms) and bridging (*via* the carboxyl­ate O atom), are exhibited by the tyrosinates. Three inter­ligand (intra­cluster) N—H⋯O hydrogen-bonding inter­actions stabilize the incomplete cubane-type moiety. Additional N—H⋯O, O—H⋯O and C—H⋯O inter­actions are formed between clusters, and between the clusters and methanol mol­ecules to regulate the spatial orientation of the tyrosinate and the assembly of the clusters in the crystal. The approximate equilateral triangular arrangement of the three nickel cations in the incomplete cubane-type moiety suggests the possible magnetic frustration, and the proximity of these metal cations indicates weak metallic bonds. The structure contains approximately 39% solvent-accessible volume between the clusters. This is filled with 17 mol­ecules of disordered methanol and was modelled with *SQUEEZE* [Spek (2009 ▶). *Acta Cryst*. D**65**, 148–155]; the reported unit-cell characteristics do not take these mol­ecules into account. The H atoms of the solvent mol­ecules have not been included in the crystal data.

## Related literature

For related incomplete cubane clusters, see: Ama *et al.* (2000 ▶); Lalia-Kantouri *et al.* (2010 ▶). For a nickel complex with l-tyrosine, see: Pei & Wang (2006 ▶). For structures with tyrosinate, see: Wojciechowska *et al.* (2011 ▶, 2012 ▶). For assignment of topology, see: Blatov (2012 ▶). For background to magnetic frustration, see: Hendrickson *et al.* (2005 ▶); Nakatsuji *et al.* (2005 ▶). For the CSD, see: Allen (2002 ▶).
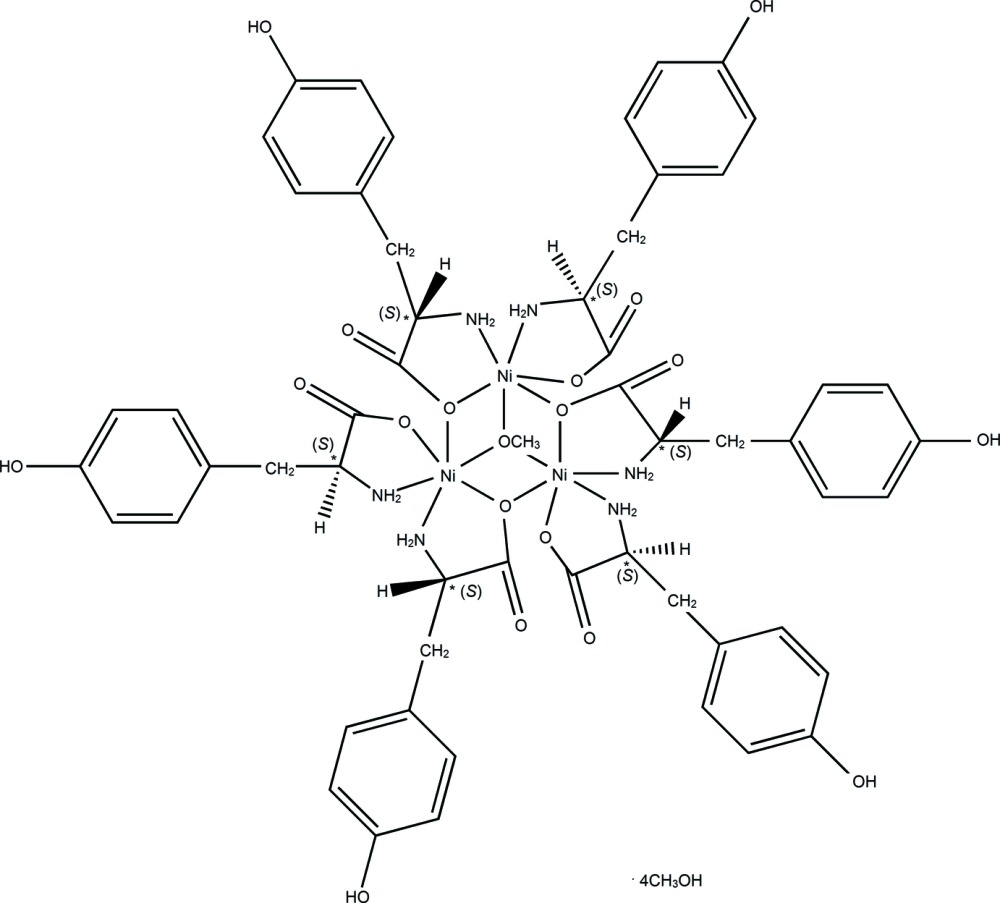


## Experimental

### 

#### Crystal data

[Ni_3_(C_9_H_10_NO_3_)_6_(CH_3_O)]·4CH_4_O
*M* *_r_* = 1400.28
Monoclinic, 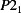

*a* = 12.5688 (6) Å
*b* = 25.3381 (9) Å
*c* = 13.1058 (7) Å
β = 96.740 (4)°
*V* = 4145.0 (3) Å^3^
*Z* = 2
Mo *K*α radiation
μ = 0.74 mm^−1^
*T* = 150 K
0.36 × 0.35 × 0.34 mm

#### Data collection

Stoe IPDS2 diffractometer
Absorption correction: analytical (a face-indexed absorption correction was applied using the Tompa method; Meulenaer de & Tompa, 1965 ▶) *T*_min_ = 0.716, *T*_max_ = 0.780
41350 measured reflections
16565 independent reflections
11845 reflections with *I* > 2σ(*I*)
*R*_int_ = 0.074

#### Refinement

*R*[*F* ^2^ > 2σ(*F* ^2^)] = 0.040
*wR*(*F* ^2^) = 0.097
*S* = 0.88
16565 reflections
780 parameters
1 restraint
H-atom parameters constrained
Δρ_max_ = 0.83 e Å^−3^
Δρ_min_ = −0.38 e Å^−3^
Absolute structure: Flack (1983 ▶), 8080 Friedel pairs
Flack parameter: 0.023 (9)

### 

Data collection: *X-AREA* (Stoe & Cie, 2002 ▶); cell refinement: *X-AREA*; data reduction: *X-AREA*; program(s) used to solve structure: *SHELXS97* (Sheldrick, 2008 ▶); program(s) used to refine structure: *SHELXL97* (Sheldrick, 2008 ▶); molecular graphics: *DIAMOND* (Brandenburg, 2006 ▶); software used to prepare material for publication: *publCIF* (Westrip, 2010 ▶).

## Supplementary Material

Crystal structure: contains datablock(s) I, global. DOI: 10.1107/S1600536813010696/tk5221sup1.cif

Structure factors: contains datablock(s) I. DOI: 10.1107/S1600536813010696/tk5221Isup2.hkl

Additional supplementary materials:  crystallographic information; 3D view; checkCIF report

## Figures and Tables

**Table 1:** Hydrogen-bond geometry (Å, °)

*D*—H⋯*A*	*D*—H	H⋯*A*	*D*⋯*A*	*D*—H⋯*A*
N1—H1*A*⋯O15^i^	0.90	2.26	3.082 (4)	153
N1—H1*B*⋯O18^i^	0.90	2.20	3.042 (4)	157
O3—H3⋯O3*M*	0.82	1.79	2.601 (5)	170
O6—H6*A*⋯O14^ii^	0.82	1.88	2.685 (4)	166
N3—H3*C*⋯O13	0.90	2.36	3.174 (4)	150
O9—H9*A*⋯O2^iii^	0.82	1.78	2.597 (4)	173
N4—H4*B*⋯O17	0.90	2.29	3.050 (5)	142
O12—H12⋯O2*M* ^iii^	0.82	1.90	2.669 (7)	155
N5—H5*A*⋯O1	0.90	2.46	3.251 (4)	146
N5—H5*A*⋯O9^iv^	0.90	2.54	3.216 (4)	133
O15—H15*A*⋯O8^v^	0.82	2.02	2.815 (4)	164
O15—H15*A*⋯O7^v^	0.82	2.47	2.983 (4)	122
O18—H18*A*⋯O11^v^	0.82	1.84	2.638 (4)	163
C1*M*—H1*M*1⋯O1	0.96	2.52	3.042 (4)	114
C30—H30*A*⋯O11	0.97	2.55	2.893 (5)	101
C38—H38⋯O9^iv^	0.98	2.38	3.178 (5)	138
C54—H54⋯O5	0.93	2.42	3.338 (5)	167

Symmetry codes: (i) 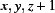; (ii) 
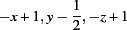
; (iii) 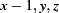; (iv) 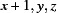; (v) 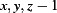.

**Fig. 1.**
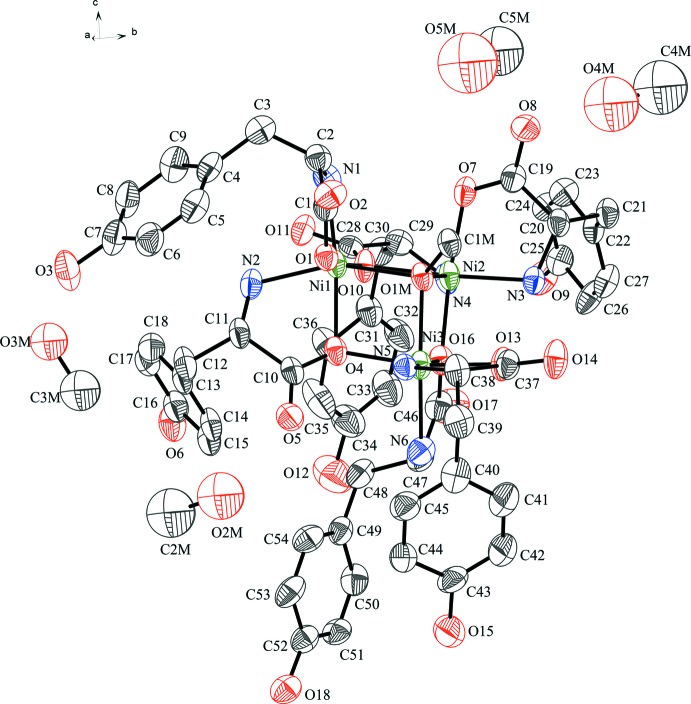
The asymmetric unit of 1 showing atom-labeling scheme and with 50% probability displacement ellipsoids. Hydrogen atoms are omitted for clarity.

**Fig. 2.**
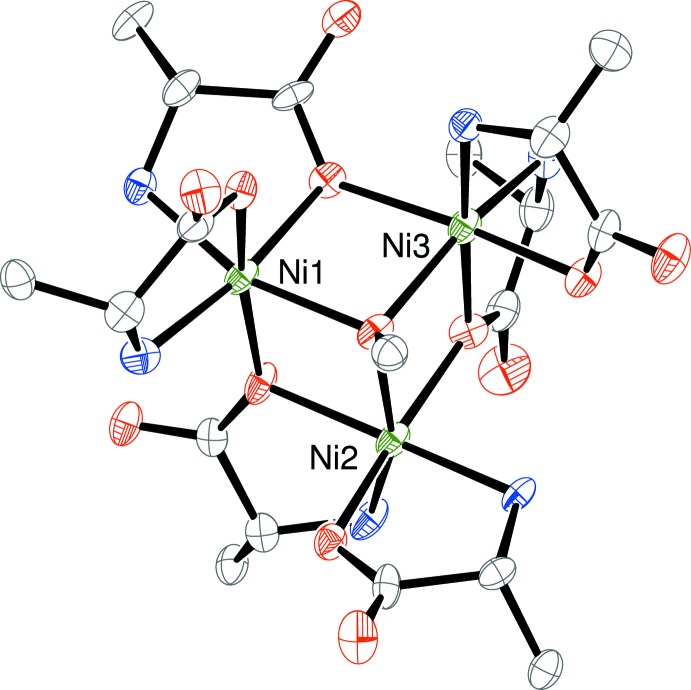
The incomplete cubane core of 1. Only selected atoms from the ligands are drawn. Atoms are shown as 30% probability ellipsoids.

**Fig. 3.**
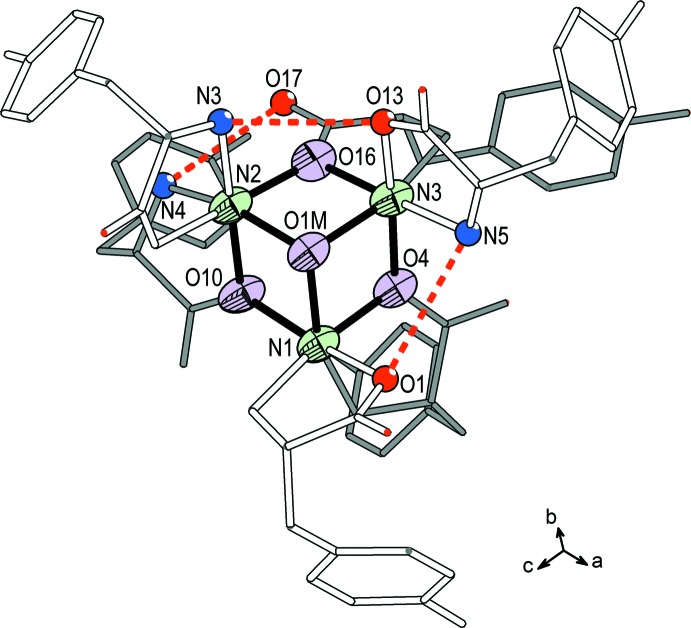
View of intra-cluster hydrogen bonding interactions (dash lines), showing the donor and acceptor atoms in a ball-and-stick model and with 50% probability ellipsoids for the incomplete cubane core. Weak C—H···O hydrogen bonding interactions are omitted. [Symmetry codes: (i) 1 - x, -1/2 + y, 1 - z (ii) -1 + x, y, z (iii) 1 + x, y, z.]

**Fig. 4.**
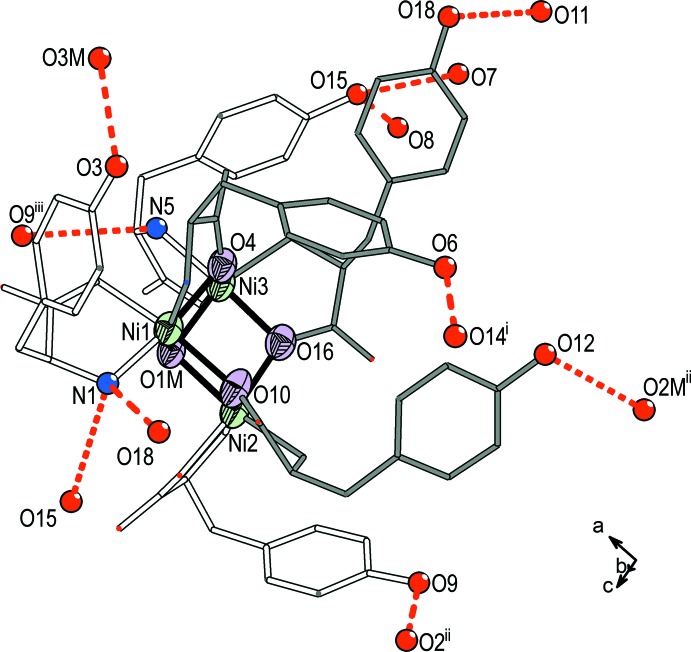
View of inter-cluster hydrogen bonding interactions (dash lines), showing the donor and acceptor atoms in a ball-and-stick model and with 50% probability ellipsoids for the incomplete cubane core. Weak C—H···O hydrogen bonding interactions are omitted. [Symmetry codes: (i) 1 - x, -1/2 + y, 1 - z (ii) -1 + x, y, z (iii) 1 + x, y, z.]

## supplementary crystallographic information

### Comment

In the investigation of anti-bacterial and anti-fungal activities of the divalent metal complexes using (*S*)-2-amino-2-methyl-3-(4'-hydroxyphenyl)propanoic acid (*L*-tyrosine) under basic conditions, single crystals of [Ni_3_(C_9_H_11_NO_3_)_6_(OCH_3_)].4CH_3_OH (1) were prepared and isolated.

The asymmetric unit of 1 contains a neutral cluster of ninety-one non-hydrogen atoms comprising three Ni ions, six tyrosinate ligands, a methoxide ion and four methanol molecules (Fig. 1). The three Ni ions adopt similar octahedral coordination geometries, completed by two monodentate amino N atoms, one monodentate carboxylate O atom, two carboxylate bridging µ_2_-O atoms, and one bridging µ_3_-O atom of the methoxide ion: {Ni(µ_1_-O)(µ_2_-O)_2_(µ_3_-O)((µ_1_-N)_2_}. The six tyrosinate ligands exhibit the common chelating mode of coordination, using the amino N atoms and either the carboxylate µ_2_-η^2^:η^0^ O atoms (O4, O10, O16) or the carboxylate µ_1_-η^1^:η^0^ O atoms (O1, O7, O13). These generate two five-membered chelate rings about each Ni center. These coordination modes are commonly found in the tyrosinate ligands (Pei & Wang, 2006; Wojciechowska *et al.*, 2011, 2012). Curiously, none of the phenolic groups of the tyrosine ligands within 1 are coordinated to the metal, despite conditions sufficiently basic to produce methoxide. The presence of the coordinated methoxide should invalidate any assumption on the presence of any extra-framework species with positive charges. The positive charge of Ni ions is therefore balanced by six tyrosinate ligands and one methoxide ion, resulting in the mean oxidation state of each nickel to be +2.33 (possibly a combination of two Ni^II^ and one Ni^III^). The solid conclusion may be derived by a magnetic study of 1.

The three {Ni(µ_1_-O)(µ_2_-O)_2_(µ_3_-O)((µ_1_-N)_2_} octahedra are condensed by edge-sharing and the addition of a µ_3_-OCH_3_ group (O1*M*) results in a trinuclear cluster with the {Ni_3_(µ_1_-O)(µ_2_-O)_2_(µ_3_-O)} incomplete cubane core (Fig. 2) (Ama *et al.*, 2000; Lalia-Kantouri *et al.*, 2010). Notably, the nickel ions present within the cluster must display a total positive charge of +7 to balanced the six tyrosinate ions and one methoxide ion. This corresponds to a mean oxidation state for the nickel of +2.33. The complete cubane core [Ni_4_O_4_] is rather well illustrated within the CSD (Allen, 2002) with over 100 examples. There are only 10 examples of the incomplete cubane core [Ni_3_O_4_]; the core within 1 is rather more symmetric than many of these examples. If the Ni atoms are taken as nodes, the{Ni_3_(µ_1_-O)(µ_2_-O)_2_(µ_3_-O)} core can be characterized as the 2-connected uninodal net of 2M3–1 topology with the vertex symbol [3] (Blatov, 2012).

The summation of the inner angles for each quadrilateral face of the {Ni_3_(µ_1_-O)(µ_2_-O)_2_(µ_3_-O)} core, *i.e.* {Ni1—O10—Ni2—O1*M*}, {Ni1—O4—Ni3—O1*M*} and {Ni2—O16—Ni3—O1*M*}, of *ca*. 360° suggest the planarity of these faces. Distributions of the Ni—µ_3_-O1*M* distances and the Ni—µ_3_-O1M—Ni angles in the ranges 2.067 (2) - 2.109 (2) Å and 97.17 (10) - 99.59 (10)°, respectively, imply an asymmetrical arrangement of the three Ni atoms about the apical µ_3_-O1*M* atom. This is also evident from the distances between any two Ni ions which vary within the range 3.132 (1) - 3.174 (1) Å. These relatively short distances between pairs of Ni cations may signal the presence of weak metallic bonds within 1. In nickel metal the Ni—Ni distance is 2.49 Å, while in the CSD Ni to Ni distances lie in the range 2.194 - 3.441 Å (Allen, 2002). The triangular arrangement of Ni ions within the cluster may also induce spin disorder and be associated with magnetic frustration. (Hendrickson *et al.*, 2005; Lalia-Kantouri *et al.*, 2010; Nakatsuji *et al.*, 2005).

According to previous literature, three inter-ligand (intra-cluster) hydrogen bonding interactions of N—H···O type were reported to be important in stabilizing the incomplete cubane structure (Ama *et al.*, 2000). This seems to be partially true for the {Ni_3_(µ_1_-O)(µ_2_-O)_2_(µ_3_-O)} core in 1, in which three N—H···O hydrogen bonding interactions, *i.e.* N3—H3C···O13, N4—H4B···O17 and N5—H5A···O1 [N···O 3.050 (5) - 3.251 (4) Å, N—H···O 142° - 150°], are present (Fig. 3). Atoms N1 and N5, in addition, reinforce the stability of the {Ni_3_(µ_1_-O)(µ_2_-O)_2_(µ_3_-O)} core *via* the inter-cluster interactions, *i.e.* N1—H1A···O15, N1—H1B···O18 and N5—H5A···O9 [N···O 3.042 (4) - 3.216 (4) Å, N—H···O 132.69° - 156.47°] (Fig. 4). The presence of the —CH_2_— group in the structure of the tyrosinate provides flexibility in spatial arrangement of the —(C_6_H_4_)OH part, depending on the surrounding environment. In the crystal structure of 1, the arrangement of these motifs is regulated by the strong O—H···O hydrogen bonding interactions (Fig. 4). The OH groups of all tyrosinate anions are associated in the O—H···O interactions with the neighboring clusters and methanol molecules [O—H···O 2.597 (4) - 2.983 (4) Å, O—H···O 122 - 173°], and these generate the supramolecular assembly in 1. The arrangement of these clusters occurs in such a way to maximize the hydrogen bonding interactions of which the weak hydrogen bonding of C—H···O type are also present, *i.e.* the intra-cluster C30—H30A···O11 and C54—H54···O5, and the inter-cluster C1M—H1M1···O1 and C38—H38···O9.

The clusters are arranged by the 2_1_ screw axis into layers in the *xz* plane. These layers are stacked in an ABAB arrangement parallel to *b*. There exist hydrogen bonds between the clusters, both within the layers and between them. This packing arrangement of clusters is rather inefficient and the structure contains large voids centred on the origin such that approximately 39% of the structure is solvent accessible volume. Methanol molecules within these regions were poorly located and the reflection data were treated with the SQUEEZE algorithm (Spek, 2009) to model electron density within these regions. These calculations reveal that each void contains around 279 electrons consistent with around 17 molecules of methanol, giving an overall composition for 1 of [Ni_3_(C_9_H_11_NO_3_)_6_(OCH_3_)].21CH_3_OH. The methanol is lost very quickly when crystals are removed from solvent and this has prevented extensive analysis of the properties of 1.

The presence of methoxide suggests that it should be possible to obtain similar structures with other weakly coordinating anions. Similarly, replacement of method by other, bulkier and less volatile solvents, may enable further studies on similar compounds, in particular magnetic measurements. Clusters of this type therefore may be suitable for fundamental magnetic studies by variation of ligand bulk, or may prove suitable nodes in the construction of framework solids by appropriate ligand choice.

### Experimental

Ni(NO_3_)_2_.6H_2_O (0.0148 g, 0.5 mmol; 98% Alfa Aesar) and 4-(HO)C_6_H_4_CH_2_CH(NH_2_)CO_2_H (*L*-tyrosine; 0.0185 g, 0.10 mmol; 98% Sigma-Aldrich) were dissolved in methanol (5.0 cm^3^; 99.8% Fisher Scientific) using a glass vial (vial A). A few drops of HCl (37% Fisher Scientific) were necessary to completely dissolve the *L*-Tyr. To a smaller glass vial, *ca*. 0.2 cm^3^ of (C_2_H_5_)_3_N (triethylamine; 99% Fisher Scientific) was added and the vial closed using lid with a small pin hole (vial B). Vial B was then inserted in vial A, which was then closed tightly. After *ca* 4 months, a few blue blocks crystallized from the solution, and were isolated for X-ray diffraction data collection.

### Refinement

The O-, N- and C-bound H-atoms were placed in calculated positions [O—H = 0.82 Å, N—H = 0.90 Å and C—H = 0.93 to 0.98 Å, *U*_iso_(H) = 1.2–1.5*U*_eq_(O, N and C)] and were included in the refinement in the riding model approximation. In order to take the contribution of the disordered methanol into account, the 'SQUEEZE option' in the program *PLATON* (Spek, 2009) was implemented. This resulted in an improvement of the *R* and *wR* from 0.073 and 0.212 to 0.040 and 0.097, respectively.

### Crystal data

**Table:**

[Ni_3_(C_9_H_10_NO_3_)_6_(CH_3_O)]·4CH_4_O	*F*(000) = 1454
*M**_r_* = 1400.28	*D*_x_ = 1.122 Mg m^−^^3^
Monoclinic, *P*2_1_	Mo *K*α radiation, λ = 0.71073 Å
Hall symbol: P 2yb	Cell parameters from 3495 reflections
*a* = 12.5688 (6) Å	θ = 1.6–28.3°
*b* = 25.3381 (9) Å	µ = 0.74 mm^−^^1^
*c* = 13.1058 (7) Å	*T* = 150 K
β = 96.740 (4)°	Block, light-blue
*V* = 4145.0 (3) Å^3^	0.36 × 0.35 × 0.34 mm
*Z* = 2	

### Data collection

**Table:**

Stoe IPDS2 diffractometer	16565 independent reflections
Radiation source: fine-focus sealed tube	11845 reflections with *I* > 2σ(*I*)
Graphite monochromator	*R*_int_ = 0.074
Detector resolution: 6.67 pixels mm^-1^	θ_max_ = 26.1°, θ_min_ = 1.6°
ω scans	*h* = −15→15
Absorption correction: analytical (a face-indexed absorption correction was applied using the Tompa method; Meulenaer de & Tompa, 1965)	*k* = −31→31
*T*_min_ = 0.716, *T*_max_ = 0.780	*l* = −16→14
41350 measured reflections	

### Refinement

**Table:**

Refinement on *F*^2^	Secondary atom site location: difference Fourier map
Least-squares matrix: full	Hydrogen site location: inferred from neighbouring sites
*R*[*F*^2^ > 2σ(*F*^2^)] = 0.040	H-atom parameters constrained
*wR*(*F*^2^) = 0.097	*w* = 1/[σ^2^(*F*_o_^2^) + (0.0444*P*)^2^] where *P* = (*F*_o_^2^ + 2*F*_c_^2^)/3
*S* = 0.88	(Δ/σ)_max_ = 0.002
16565 reflections	Δρ_max_ = 0.83 e Å^−^^3^
780 parameters	Δρ_min_ = −0.38 e Å^−^^3^
1 restraint	Absolute structure: Flack (1983), 8080 Friedel pairs
Primary atom site location: structure-invariant direct methods	Flack parameter: 0.023 (9)

### Special details

**Table:**

Geometry. All e.s.d.'s (except the e.s.d. in the dihedral angle between two l.s. planes) are estimated using the full covariance matrix. The cell e.s.d.'s are taken into account individually in the estimation of e.s.d.'s in distances, angles and torsion angles; correlations between e.s.d.'s in cell parameters are only used when they are defined by crystal symmetry. An approximate (isotropic) treatment of cell e.s.d.'s is used for estimating e.s.d.'s involving l.s. planes.
Refinement. Refinement of *F*^2^ against ALL reflections. The weighted *R*-factor *wR* and goodness of fit *S* are based on *F*^2^, conventional *R*-factors *R* are based on *F*, with *F* set to zero for negative *F*^2^. The threshold expression of *F*^2^ > σ(*F*^2^) is used only for calculating *R*-factors(gt) *etc*. and is not relevant to the choice of reflections for refinement. *R*-factors based on *F*^2^ are statistically about twice as large as those based on *F*, and *R*- factors based on ALL data will be even larger.

### Fractional atomic coordinates and isotropic or equivalent isotropic displacement parameters (Å^2^)

**Table:**

	*x*	*y*	*z*	*U*_iso_*/*U*_eq_	
Ni1	0.67401 (3)	0.209152 (16)	0.66058 (4)	0.03463 (11)	
O1M	0.64605 (17)	0.28831 (10)	0.62163 (19)	0.0350 (5)	
N1	0.7077 (2)	0.21031 (14)	0.8182 (2)	0.0432 (7)	
H1A	0.6691	0.2359	0.8440	0.052*	
H1B	0.6884	0.1793	0.8439	0.052*	
O1	0.83540 (19)	0.22349 (9)	0.6684 (2)	0.0369 (6)	
C1	0.8803 (3)	0.23246 (14)	0.7588 (3)	0.0404 (9)	
O2	0.9740 (2)	0.25142 (11)	0.7761 (2)	0.0483 (7)	
C2	0.8238 (3)	0.21973 (14)	0.8507 (3)	0.0426 (9)	
H2	0.8296	0.2507	0.8958	0.051*	
C3	0.8785 (3)	0.17307 (16)	0.9118 (3)	0.0477 (10)	
H3A	0.9498	0.1841	0.9403	0.057*	
H3B	0.8385	0.1655	0.9691	0.057*	
C4	0.8886 (3)	0.12271 (16)	0.8527 (3)	0.0478 (10)	
C5	0.9647 (3)	0.11715 (17)	0.7819 (4)	0.0558 (11)	
H5	1.0082	0.1459	0.7713	0.067*	
C6	0.9773 (4)	0.07274 (18)	0.7297 (4)	0.0614 (12)	
H6	1.0295	0.0707	0.6851	0.074*	
C7	0.9103 (4)	0.02862 (17)	0.7426 (4)	0.0590 (12)	
C8	0.8360 (4)	0.03291 (17)	0.8129 (4)	0.0612 (13)	
H8	0.7916	0.0046	0.8234	0.073*	
C9	0.8280 (3)	0.07957 (17)	0.8676 (4)	0.0567 (11)	
H9	0.7795	0.0813	0.9159	0.068*	
O3	0.9161 (3)	−0.01815 (12)	0.6914 (3)	0.0763 (10)	
H3	0.9644	−0.0166	0.6545	0.114*	
N2	0.6949 (2)	0.12825 (11)	0.6396 (3)	0.0416 (8)	
H2A	0.7321	0.1140	0.6957	0.050*	
H2B	0.6310	0.1120	0.6282	0.050*	
O4	0.66026 (18)	0.20168 (9)	0.5025 (2)	0.0370 (6)	
C10	0.7201 (3)	0.16670 (13)	0.4722 (3)	0.0363 (9)	
O5	0.7561 (2)	0.16689 (11)	0.3872 (2)	0.0455 (6)	
C11	0.7544 (3)	0.12247 (13)	0.5498 (3)	0.0405 (9)	
H11	0.8301	0.1285	0.5743	0.049*	
C12	0.7462 (3)	0.06642 (14)	0.5046 (4)	0.0524 (11)	
H12A	0.7801	0.0660	0.4418	0.063*	
H12B	0.7859	0.0425	0.5527	0.063*	
C13	0.6346 (3)	0.04650 (13)	0.4814 (3)	0.0420 (9)	
C14	0.5666 (3)	0.06193 (14)	0.3962 (4)	0.0467 (10)	
H14	0.5922	0.0858	0.3509	0.056*	
C15	0.4626 (3)	0.04394 (14)	0.3745 (4)	0.0473 (10)	
H15	0.4191	0.0556	0.3166	0.057*	
C16	0.4246 (3)	0.00790 (15)	0.4418 (3)	0.0476 (10)	
C17	0.4897 (3)	−0.00772 (15)	0.5270 (3)	0.0469 (10)	
H17	0.4648	−0.0318	0.5723	0.056*	
C18	0.5934 (3)	0.01222 (16)	0.5464 (3)	0.0501 (10)	
H18	0.6360	0.0018	0.6059	0.060*	
O6	0.3242 (2)	−0.01120 (10)	0.4159 (2)	0.0552 (8)	
H6A	0.3093	−0.0318	0.4604	0.083*	
Ni2	0.48220 (3)	0.291099 (15)	0.62530 (4)	0.03586 (11)	
N3	0.4712 (2)	0.37166 (11)	0.6007 (2)	0.0358 (7)	
H3C	0.5228	0.3820	0.5629	0.043*	
H3D	0.4074	0.3794	0.5653	0.043*	
O7	0.4971 (2)	0.31369 (9)	0.7749 (2)	0.0407 (6)	
C19	0.5061 (3)	0.36282 (14)	0.7897 (3)	0.0404 (9)	
O8	0.5333 (3)	0.38163 (11)	0.8774 (2)	0.0601 (8)	
C20	0.4830 (3)	0.40097 (13)	0.7005 (3)	0.0368 (8)	
H20	0.5445	0.4248	0.7010	0.044*	
C21	0.3828 (3)	0.43479 (14)	0.7125 (3)	0.0423 (9)	
H21A	0.3888	0.4487	0.7819	0.051*	
H21B	0.3813	0.4645	0.6658	0.051*	
C22	0.2789 (3)	0.40506 (13)	0.6917 (3)	0.0390 (9)	
C23	0.2428 (3)	0.37308 (15)	0.7650 (3)	0.0451 (9)	
H23	0.2813	0.3720	0.8301	0.054*	
C24	0.1507 (3)	0.34206 (15)	0.7456 (3)	0.0434 (9)	
H24	0.1278	0.3210	0.7969	0.052*	
C25	0.0942 (3)	0.34335 (15)	0.6479 (3)	0.0428 (9)	
C26	0.1283 (3)	0.37557 (17)	0.5735 (4)	0.0528 (11)	
H26	0.0900	0.3769	0.5084	0.063*	
C27	0.2194 (3)	0.40591 (15)	0.5956 (4)	0.0479 (10)	
H27	0.2415	0.4275	0.5447	0.058*	
O9	0.0065 (2)	0.31212 (11)	0.6231 (2)	0.0548 (7)	
H9A	−0.0056	0.2951	0.6737	0.082*	
N4	0.3258 (2)	0.26513 (11)	0.6264 (3)	0.0389 (7)	
H4A	0.2840	0.2915	0.6447	0.047*	
H4B	0.2991	0.2535	0.5637	0.047*	
O10	0.50697 (18)	0.21246 (10)	0.6586 (2)	0.0396 (6)	
C28	0.4328 (3)	0.18951 (14)	0.6989 (3)	0.0360 (8)	
O11	0.4391 (2)	0.14462 (10)	0.7387 (2)	0.0448 (6)	
C29	0.3300 (3)	0.22191 (14)	0.7017 (3)	0.0394 (9)	
H29	0.3360	0.2387	0.7695	0.047*	
C30	0.2260 (3)	0.18813 (15)	0.6932 (3)	0.0425 (9)	
H30A	0.2380	0.1583	0.7394	0.051*	
H30B	0.1693	0.2093	0.7166	0.051*	
C31	0.1882 (3)	0.16788 (15)	0.5888 (3)	0.0462 (10)	
C32	0.0857 (3)	0.18068 (18)	0.5424 (4)	0.0610 (13)	
H32	0.0437	0.2038	0.5756	0.073*	
C33	0.0455 (4)	0.16009 (19)	0.4494 (4)	0.0635 (13)	
H33	−0.0248	0.1676	0.4228	0.076*	
C34	0.1067 (4)	0.1288 (2)	0.3951 (4)	0.0661 (13)	
C35	0.2115 (4)	0.1169 (2)	0.4365 (5)	0.0740 (16)	
H35	0.2556	0.0973	0.3986	0.089*	
C36	0.2488 (3)	0.13418 (18)	0.5326 (4)	0.0577 (12)	
H36	0.3163	0.1234	0.5618	0.069*	
O12	0.0715 (3)	0.10594 (17)	0.3024 (3)	0.0922 (12)	
H12	0.0108	0.1164	0.2828	0.138*	
Ni3	0.64300 (3)	0.280740 (15)	0.46105 (4)	0.03642 (11)	
N5	0.8050 (2)	0.28923 (12)	0.4539 (2)	0.0386 (7)	
H5A	0.8411	0.2779	0.5133	0.046*	
H5B	0.8243	0.2689	0.4029	0.046*	
O13	0.64711 (19)	0.36165 (10)	0.4467 (2)	0.0436 (7)	
C37	0.7391 (3)	0.38049 (14)	0.4409 (3)	0.0422 (9)	
O14	0.7563 (2)	0.42996 (10)	0.4409 (3)	0.0553 (8)	
C38	0.8346 (3)	0.34438 (14)	0.4360 (3)	0.0437 (10)	
H38	0.8876	0.3543	0.4937	0.052*	
C39	0.8885 (3)	0.35072 (18)	0.3408 (4)	0.0565 (11)	
H39A	0.9092	0.3874	0.3347	0.068*	
H39B	0.9534	0.3297	0.3476	0.068*	
C40	0.8211 (3)	0.33499 (17)	0.2459 (4)	0.0558 (11)	
C41	0.7431 (4)	0.3691 (2)	0.1951 (4)	0.0692 (14)	
H41	0.7337	0.4022	0.2234	0.083*	
C42	0.6809 (4)	0.3557 (2)	0.1066 (4)	0.0690 (13)	
H42	0.6305	0.3795	0.0763	0.083*	
C43	0.6922 (3)	0.30699 (17)	0.0614 (3)	0.0530 (11)	
C44	0.7645 (4)	0.27061 (18)	0.1084 (3)	0.0602 (12)	
H44	0.7697	0.2370	0.0807	0.072*	
C45	0.8300 (3)	0.2852 (2)	0.1989 (3)	0.0555 (10)	
H45	0.8807	0.2613	0.2285	0.067*	
O15	0.6373 (3)	0.29244 (14)	−0.0297 (2)	0.0757 (9)	
H15A	0.5968	0.3163	−0.0512	0.114*	
N6	0.5834 (3)	0.26790 (13)	0.3064 (3)	0.0452 (8)	
H6B	0.5877	0.2980	0.2706	0.054*	
H6C	0.6232	0.2432	0.2793	0.054*	
O16	0.48472 (19)	0.27843 (10)	0.4722 (2)	0.0409 (6)	
C46	0.4195 (3)	0.26377 (15)	0.3922 (3)	0.0444 (9)	
O17	0.3229 (2)	0.26011 (14)	0.3935 (3)	0.0698 (9)	
C47	0.4695 (3)	0.25007 (15)	0.2987 (3)	0.0442 (9)	
H47	0.4292	0.2676	0.2395	0.053*	
C48	0.4619 (3)	0.18945 (15)	0.2818 (3)	0.0463 (10)	
H48A	0.5085	0.1722	0.3361	0.056*	
H48B	0.3891	0.1783	0.2880	0.056*	
C49	0.4916 (3)	0.17103 (14)	0.1804 (3)	0.0416 (9)	
C50	0.4162 (3)	0.17076 (15)	0.0914 (3)	0.0483 (10)	
H50	0.3466	0.1820	0.0961	0.058*	
C51	0.4432 (3)	0.15440 (15)	−0.0011 (3)	0.0452 (9)	
H51	0.3914	0.1545	−0.0580	0.054*	
C52	0.5463 (3)	0.13756 (15)	−0.0125 (3)	0.0435 (9)	
C53	0.6209 (3)	0.13709 (17)	0.0737 (3)	0.0522 (11)	
H53	0.6900	0.1252	0.0684	0.063*	
C54	0.5945 (3)	0.15397 (16)	0.1677 (3)	0.0481 (10)	
H54	0.6469	0.1539	0.2240	0.058*	
O18	0.5789 (2)	0.12088 (11)	−0.1018 (2)	0.0493 (7)	
H18A	0.5288	0.1227	−0.1478	0.074*	
O3M	1.0553 (3)	−0.02010 (15)	0.5593 (3)	0.0815 (10)*	
C1M	0.7126 (3)	0.32667 (13)	0.6751 (3)	0.0384 (9)	
H1M1	0.7859	0.3200	0.6652	0.058*	
H1M2	0.7049	0.3250	0.7470	0.058*	
H1M3	0.6923	0.3611	0.6492	0.058*	
O2M	0.8596 (5)	0.1098 (2)	0.2522 (4)	0.1298 (17)*	
C3M	1.0306 (5)	0.0038 (3)	0.4648 (5)	0.0954 (18)*	
C2M	0.8170 (8)	0.0539 (4)	0.2325 (7)	0.140 (3)*	
O4M	0.6264 (6)	0.4805 (3)	0.9009 (6)	0.177 (3)*	
C4M	0.5490 (9)	0.5181 (5)	0.9249 (9)	0.172 (4)*	
O5M	0.3634 (7)	0.3045 (4)	1.0147 (6)	0.215 (3)*	
C5M	0.2700 (8)	0.3219 (4)	1.0342 (7)	0.142 (3)*	

### Atomic displacement parameters (Å^2^)

**Table:**

	*U*^11^	*U*^22^	*U*^33^	*U*^12^	*U*^13^	*U*^23^
Ni1	0.0304 (2)	0.0249 (2)	0.0472 (3)	−0.00070 (19)	−0.0011 (2)	−0.0033 (2)
O1M	0.0297 (11)	0.0255 (12)	0.0488 (14)	−0.0037 (11)	−0.0001 (11)	−0.0059 (13)
N1	0.0360 (15)	0.0412 (16)	0.053 (2)	−0.0085 (15)	0.0067 (14)	−0.0080 (17)
O1	0.0336 (12)	0.0317 (13)	0.0440 (16)	−0.0004 (10)	−0.0013 (12)	−0.0034 (11)
C1	0.0353 (19)	0.0343 (18)	0.050 (3)	−0.0008 (16)	−0.0009 (19)	−0.0015 (18)
O2	0.0377 (14)	0.0554 (16)	0.0489 (18)	−0.0156 (12)	−0.0075 (12)	0.0015 (13)
C2	0.0403 (18)	0.039 (2)	0.048 (2)	−0.0038 (16)	0.0012 (17)	−0.0046 (17)
C3	0.043 (2)	0.053 (2)	0.047 (3)	−0.0058 (18)	0.0026 (19)	0.005 (2)
C4	0.044 (2)	0.045 (2)	0.052 (3)	0.0069 (18)	−0.0030 (19)	0.0143 (19)
C5	0.048 (2)	0.051 (2)	0.066 (3)	0.001 (2)	−0.001 (2)	0.016 (2)
C6	0.061 (3)	0.060 (3)	0.065 (3)	0.022 (2)	0.014 (2)	0.008 (2)
C7	0.062 (3)	0.043 (2)	0.072 (3)	0.015 (2)	0.007 (2)	0.014 (2)
C8	0.068 (3)	0.039 (2)	0.079 (4)	0.011 (2)	0.018 (3)	0.014 (2)
C9	0.043 (2)	0.054 (2)	0.075 (3)	0.0042 (19)	0.016 (2)	0.011 (2)
O3	0.092 (2)	0.0403 (16)	0.098 (3)	0.0149 (17)	0.017 (2)	−0.0075 (17)
N2	0.0390 (16)	0.0276 (15)	0.055 (2)	−0.0005 (13)	−0.0062 (16)	0.0028 (15)
O4	0.0306 (12)	0.0285 (13)	0.0505 (16)	−0.0010 (10)	−0.0018 (11)	−0.0048 (12)
C10	0.0255 (16)	0.0301 (17)	0.049 (3)	−0.0015 (14)	−0.0118 (17)	−0.0123 (17)
O5	0.0422 (14)	0.0469 (15)	0.0460 (18)	0.0047 (12)	−0.0009 (13)	−0.0061 (13)
C11	0.0377 (19)	0.0246 (16)	0.057 (3)	0.0008 (15)	−0.0040 (18)	−0.0050 (17)
C12	0.047 (2)	0.0325 (19)	0.074 (3)	0.0084 (17)	−0.009 (2)	−0.012 (2)
C13	0.044 (2)	0.0233 (17)	0.056 (3)	0.0032 (15)	−0.004 (2)	−0.0103 (17)
C14	0.045 (2)	0.0256 (17)	0.069 (3)	0.0033 (16)	0.003 (2)	−0.0084 (18)
C15	0.039 (2)	0.0322 (19)	0.067 (3)	0.0031 (16)	−0.009 (2)	−0.0080 (19)
C16	0.046 (2)	0.0349 (19)	0.061 (3)	0.0006 (17)	0.001 (2)	−0.0070 (19)
C17	0.056 (2)	0.0332 (19)	0.051 (3)	−0.0023 (18)	0.001 (2)	−0.0068 (18)
C18	0.049 (2)	0.042 (2)	0.055 (3)	0.0013 (18)	−0.013 (2)	−0.004 (2)
O6	0.0456 (15)	0.0383 (14)	0.079 (2)	−0.0074 (12)	−0.0034 (15)	−0.0070 (14)
Ni2	0.0298 (2)	0.0241 (2)	0.0525 (3)	−0.00098 (18)	0.0001 (2)	−0.0018 (2)
N3	0.0310 (15)	0.0288 (15)	0.046 (2)	0.0016 (12)	−0.0017 (14)	0.0017 (14)
O7	0.0397 (13)	0.0277 (12)	0.0528 (17)	−0.0013 (10)	−0.0032 (12)	0.0049 (11)
C19	0.0345 (18)	0.035 (2)	0.049 (3)	−0.0052 (15)	−0.0072 (18)	0.0006 (18)
O8	0.087 (2)	0.0409 (14)	0.0453 (18)	−0.0113 (15)	−0.0201 (16)	−0.0072 (14)
C20	0.0369 (19)	0.0251 (16)	0.047 (2)	−0.0045 (15)	−0.0018 (17)	−0.0022 (16)
C21	0.040 (2)	0.0293 (17)	0.055 (3)	−0.0007 (15)	−0.0047 (18)	−0.0007 (17)
C22	0.0329 (18)	0.0289 (17)	0.055 (3)	0.0049 (15)	0.0039 (18)	0.0020 (17)
C23	0.038 (2)	0.049 (2)	0.047 (3)	−0.0048 (17)	0.0014 (19)	0.0023 (19)
C24	0.0348 (18)	0.048 (2)	0.046 (2)	−0.0067 (17)	0.0018 (18)	0.0051 (18)
C25	0.0280 (17)	0.046 (2)	0.054 (3)	0.0023 (16)	0.0039 (18)	0.0057 (19)
C26	0.036 (2)	0.054 (2)	0.065 (3)	0.0026 (18)	−0.009 (2)	0.025 (2)
C27	0.038 (2)	0.041 (2)	0.063 (3)	0.0026 (17)	0.001 (2)	0.017 (2)
O9	0.0318 (13)	0.0611 (17)	0.069 (2)	−0.0089 (12)	−0.0054 (13)	0.0149 (15)
N4	0.0329 (15)	0.0278 (14)	0.056 (2)	−0.0001 (12)	0.0029 (14)	−0.0076 (14)
O10	0.0294 (11)	0.0236 (11)	0.0647 (18)	−0.0029 (11)	0.0007 (12)	0.0042 (13)
C28	0.0399 (19)	0.0334 (18)	0.032 (2)	−0.0048 (15)	−0.0058 (16)	−0.0072 (16)
O11	0.0424 (14)	0.0324 (13)	0.0554 (18)	−0.0034 (11)	−0.0123 (13)	0.0062 (12)
C29	0.0352 (18)	0.041 (2)	0.041 (2)	−0.0084 (15)	−0.0010 (16)	−0.0060 (16)
C30	0.0379 (19)	0.0405 (19)	0.048 (3)	−0.0036 (16)	−0.0008 (18)	0.0030 (18)
C31	0.038 (2)	0.0355 (19)	0.063 (3)	−0.0091 (17)	−0.001 (2)	−0.0093 (19)
C32	0.049 (2)	0.059 (3)	0.071 (3)	0.015 (2)	−0.010 (2)	−0.023 (2)
C33	0.051 (2)	0.057 (3)	0.082 (4)	0.006 (2)	0.001 (3)	−0.014 (3)
C34	0.060 (3)	0.068 (3)	0.067 (3)	0.003 (2)	−0.009 (2)	−0.021 (3)
C35	0.050 (3)	0.064 (3)	0.106 (4)	0.004 (2)	0.001 (3)	−0.040 (3)
C36	0.033 (2)	0.059 (3)	0.077 (3)	0.0037 (19)	−0.011 (2)	−0.029 (2)
O12	0.081 (3)	0.101 (3)	0.090 (3)	0.010 (2)	−0.006 (2)	−0.033 (2)
Ni3	0.0310 (2)	0.0278 (2)	0.0490 (3)	−0.00175 (18)	−0.0016 (2)	−0.0004 (2)
N5	0.0376 (15)	0.0345 (15)	0.0420 (17)	0.0044 (15)	−0.0027 (13)	−0.0044 (15)
O13	0.0316 (13)	0.0301 (13)	0.067 (2)	−0.0019 (11)	−0.0009 (13)	0.0051 (13)
C37	0.044 (2)	0.0297 (18)	0.052 (3)	−0.0033 (16)	0.0003 (19)	0.0033 (17)
O14	0.0427 (15)	0.0307 (13)	0.091 (2)	−0.0059 (12)	0.0022 (15)	0.0084 (14)
C38	0.0322 (18)	0.0358 (19)	0.064 (3)	−0.0066 (15)	0.0073 (19)	−0.0021 (18)
C39	0.051 (2)	0.051 (2)	0.068 (3)	−0.008 (2)	0.008 (2)	−0.003 (2)
C40	0.040 (2)	0.053 (3)	0.074 (3)	−0.0022 (19)	0.009 (2)	0.008 (2)
C41	0.077 (3)	0.060 (3)	0.068 (4)	0.013 (3)	−0.004 (3)	0.011 (3)
C42	0.078 (3)	0.060 (3)	0.064 (3)	0.022 (3)	−0.011 (3)	0.004 (3)
C43	0.052 (2)	0.063 (3)	0.040 (2)	0.010 (2)	−0.007 (2)	0.008 (2)
C44	0.073 (3)	0.055 (3)	0.052 (3)	0.023 (2)	0.003 (2)	−0.001 (2)
C45	0.054 (2)	0.062 (3)	0.049 (2)	0.008 (2)	0.0030 (19)	0.004 (2)
O15	0.084 (2)	0.072 (2)	0.065 (2)	0.028 (2)	−0.0147 (17)	−0.0137 (19)
N6	0.0452 (17)	0.0402 (17)	0.048 (2)	−0.0112 (14)	−0.0028 (15)	0.0045 (15)
O16	0.0355 (12)	0.0340 (14)	0.0521 (16)	−0.0046 (12)	0.0008 (12)	−0.0027 (13)
C46	0.0300 (19)	0.045 (2)	0.054 (3)	−0.0014 (16)	−0.0109 (18)	0.0026 (19)
O17	0.0342 (16)	0.100 (3)	0.071 (2)	−0.0017 (15)	−0.0130 (14)	0.0005 (19)
C47	0.042 (2)	0.044 (2)	0.043 (3)	−0.0003 (17)	−0.0103 (18)	0.0034 (18)
C48	0.043 (2)	0.043 (2)	0.050 (3)	−0.0106 (17)	−0.0079 (19)	0.0107 (19)
C49	0.042 (2)	0.0322 (18)	0.048 (2)	−0.0059 (16)	−0.0057 (18)	0.0027 (17)
C50	0.0361 (19)	0.045 (2)	0.061 (3)	−0.0011 (17)	−0.0061 (19)	0.000 (2)
C51	0.040 (2)	0.051 (2)	0.040 (2)	−0.0015 (17)	−0.0104 (18)	0.0021 (19)
C52	0.043 (2)	0.043 (2)	0.044 (2)	−0.0086 (17)	0.0039 (19)	0.0094 (18)
C53	0.041 (2)	0.056 (2)	0.056 (3)	−0.0042 (19)	−0.009 (2)	0.019 (2)
C54	0.039 (2)	0.059 (3)	0.044 (3)	−0.0040 (19)	−0.0073 (19)	0.007 (2)
O18	0.0405 (14)	0.0566 (16)	0.0477 (18)	−0.0053 (13)	−0.0080 (13)	0.0044 (14)
C1M	0.0333 (17)	0.0302 (18)	0.051 (2)	−0.0019 (15)	0.0035 (17)	−0.0046 (17)

### Geometric parameters (Å, º)

**Table:**

Ni1—O1	2.051 (2)	C27—H27	0.9300
Ni1—N1	2.060 (3)	O9—H9A	0.8200
Ni1—O4	2.067 (3)	N4—C29	1.471 (5)
Ni1—N2	2.089 (3)	N4—H4A	0.9000
Ni1—O1M	2.089 (3)	N4—H4B	0.9000
Ni1—O10	2.098 (2)	O10—C28	1.265 (4)
O1M—C1M	1.413 (4)	C28—O11	1.250 (4)
O1M—Ni2	2.067 (2)	C28—C29	1.535 (5)
O1M—Ni3	2.109 (2)	C29—C30	1.556 (5)
N1—C2	1.490 (5)	C29—H29	0.9800
N1—H1A	0.9000	C30—C31	1.486 (6)
N1—H1B	0.9000	C30—H30A	0.9700
O1—C1	1.272 (5)	C30—H30B	0.9700
C1—O2	1.268 (4)	C31—C32	1.397 (6)
C1—C2	1.504 (5)	C31—C36	1.409 (6)
C2—C3	1.544 (5)	C32—C33	1.367 (6)
C2—H2	0.9800	C32—H32	0.9300
C3—C4	1.506 (6)	C33—C34	1.362 (6)
C3—H3A	0.9700	C33—H33	0.9300
C3—H3B	0.9700	C34—O12	1.371 (6)
C4—C9	1.360 (6)	C34—C35	1.397 (7)
C4—C5	1.415 (6)	C35—C36	1.363 (7)
C5—C6	1.336 (6)	C35—H35	0.9300
C5—H5	0.9300	C36—H36	0.9300
C6—C7	1.421 (6)	O12—H12	0.8200
C6—H6	0.9300	Ni3—O16	2.013 (2)
C7—O3	1.368 (5)	Ni3—O13	2.060 (3)
C7—C8	1.392 (6)	Ni3—N5	2.060 (3)
C8—C9	1.392 (6)	Ni3—N6	2.102 (3)
C8—H8	0.9300	N5—C38	1.472 (5)
C9—H9	0.9300	N5—H5A	0.9000
O3—H3	0.8200	N5—H5B	0.9000
N2—C11	1.473 (5)	O13—C37	1.261 (4)
N2—H2A	0.9000	C37—O14	1.272 (4)
N2—H2B	0.9000	C37—C38	1.517 (5)
O4—C10	1.256 (4)	C38—C39	1.497 (6)
O4—Ni3	2.080 (2)	C38—H38	0.9800
C10—O5	1.251 (4)	C39—C40	1.475 (7)
C10—C11	1.541 (5)	C39—H39A	0.9700
C11—C12	1.538 (5)	C39—H39B	0.9700
C11—H11	0.9800	C40—C41	1.413 (7)
C12—C13	1.488 (5)	C40—C45	1.414 (7)
C12—H12A	0.9700	C41—C42	1.363 (7)
C12—H12B	0.9700	C41—H41	0.9300
C13—C18	1.361 (6)	C42—C43	1.383 (7)
C13—C14	1.381 (6)	C42—H42	0.9300
C14—C15	1.382 (5)	C43—O15	1.358 (5)
C14—H14	0.9300	C43—C44	1.387 (6)
C15—C16	1.392 (6)	C44—C45	1.411 (6)
C15—H15	0.9300	C44—H44	0.9300
C16—O6	1.356 (5)	C45—H45	0.9300
C16—C17	1.364 (6)	O15—H15A	0.8200
C17—C18	1.393 (6)	N6—C47	1.494 (5)
C17—H17	0.9300	N6—H6B	0.9000
C18—H18	0.9300	N6—H6C	0.9000
O6—H6A	0.8200	O16—C46	1.307 (5)
Ni2—O7	2.030 (3)	C46—O17	1.219 (4)
Ni2—O16	2.036 (3)	C46—C47	1.483 (6)
Ni2—O10	2.056 (3)	C47—C48	1.553 (5)
Ni2—N3	2.069 (3)	C47—H47	0.9800
Ni2—N4	2.074 (3)	C48—C49	1.497 (6)
N3—C20	1.496 (5)	C48—H48A	0.9700
N3—H3C	0.9000	C48—H48B	0.9700
N3—H3D	0.9000	C49—C54	1.392 (5)
O7—C19	1.263 (4)	C49—C50	1.414 (5)
C19—O8	1.255 (5)	C50—C51	1.361 (6)
C19—C20	1.518 (5)	C50—H50	0.9300
C20—C21	1.546 (5)	C51—C52	1.390 (5)
C20—H20	0.9800	C51—H51	0.9300
C21—C22	1.505 (5)	C52—O18	1.352 (5)
C21—H21A	0.9700	C52—C53	1.381 (6)
C21—H21B	0.9700	C53—C54	1.381 (6)
C22—C23	1.374 (5)	C53—H53	0.9300
C22—C27	1.387 (6)	C54—H54	0.9300
C23—C24	1.398 (5)	O18—H18A	0.8200
C23—H23	0.9300	O3M—C3M	1.382 (7)
C24—C25	1.390 (6)	C1M—H1M1	0.9600
C24—H24	0.9300	C1M—H1M2	0.9600
C25—O9	1.365 (5)	C1M—H1M3	0.9600
C25—C26	1.378 (6)	O2M—C2M	1.526 (10)
C26—C27	1.382 (6)	O4M—C4M	1.423 (12)
C26—H26	0.9300	O5M—C5M	1.306 (11)
			
O1—Ni1—N1	82.06 (11)	C25—C26—H26	120.0
O1—Ni1—O4	91.83 (10)	C27—C26—H26	120.0
N1—Ni1—O4	171.72 (11)	C26—C27—C22	121.8 (4)
O1—Ni1—N2	92.38 (11)	C26—C27—H27	119.1
N1—Ni1—N2	97.58 (14)	C22—C27—H27	119.1
O4—Ni1—N2	77.00 (12)	C25—O9—H9A	109.5
O1—Ni1—O1M	88.80 (9)	C29—N4—Ni2	106.4 (2)
N1—Ni1—O1M	103.90 (12)	C29—N4—H4A	110.5
O4—Ni1—O1M	81.44 (9)	Ni2—N4—H4A	110.5
N2—Ni1—O1M	158.43 (11)	C29—N4—H4B	110.5
O1—Ni1—O10	167.32 (10)	Ni2—N4—H4B	110.5
N1—Ni1—O10	95.69 (11)	H4A—N4—H4B	108.6
O4—Ni1—O10	91.47 (10)	C28—O10—Ni2	115.7 (2)
N2—Ni1—O10	100.29 (11)	C28—O10—Ni1	139.6 (2)
O1M—Ni1—O10	79.59 (9)	Ni2—O10—Ni1	99.64 (10)
C1M—O1M—Ni2	119.9 (2)	O11—C28—O10	125.3 (3)
C1M—O1M—Ni1	117.8 (2)	O11—C28—C29	119.3 (3)
Ni2—O1M—Ni1	99.59 (10)	O10—C28—C29	115.3 (3)
C1M—O1M—Ni3	119.8 (2)	N4—C29—C28	110.2 (3)
Ni2—O1M—Ni3	97.17 (10)	N4—C29—C30	113.5 (3)
Ni1—O1M—Ni3	98.01 (10)	C28—C29—C30	114.0 (3)
C2—N1—Ni1	111.6 (2)	N4—C29—H29	106.2
C2—N1—H1A	109.3	C28—C29—H29	106.2
Ni1—N1—H1A	109.3	C30—C29—H29	106.2
C2—N1—H1B	109.3	C31—C30—C29	115.5 (3)
Ni1—N1—H1B	109.3	C31—C30—H30A	108.4
H1A—N1—H1B	108.0	C29—C30—H30A	108.4
C1—O1—Ni1	114.0 (2)	C31—C30—H30B	108.4
O2—C1—O1	122.4 (4)	C29—C30—H30B	108.4
O2—C1—C2	117.0 (4)	H30A—C30—H30B	107.5
O1—C1—C2	120.6 (3)	C32—C31—C36	116.2 (4)
N1—C2—C1	110.1 (3)	C32—C31—C30	120.0 (4)
N1—C2—C3	112.8 (3)	C36—C31—C30	123.8 (4)
C1—C2—C3	110.8 (3)	C33—C32—C31	121.7 (4)
N1—C2—H2	107.6	C33—C32—H32	119.2
C1—C2—H2	107.6	C31—C32—H32	119.2
C3—C2—H2	107.6	C34—C33—C32	121.0 (4)
C4—C3—C2	116.2 (3)	C34—C33—H33	119.5
C4—C3—H3A	108.2	C32—C33—H33	119.5
C2—C3—H3A	108.2	C33—C34—O12	124.4 (4)
C4—C3—H3B	108.2	C33—C34—C35	119.1 (5)
C2—C3—H3B	108.2	O12—C34—C35	116.4 (4)
H3A—C3—H3B	107.4	C36—C35—C34	119.8 (4)
C9—C4—C5	116.6 (4)	C36—C35—H35	120.1
C9—C4—C3	121.6 (4)	C34—C35—H35	120.1
C5—C4—C3	121.7 (4)	C35—C36—C31	121.9 (4)
C6—C5—C4	123.3 (4)	C35—C36—H36	119.1
C6—C5—H5	118.4	C31—C36—H36	119.1
C4—C5—H5	118.4	C34—O12—H12	109.5
C5—C6—C7	119.6 (4)	O16—Ni3—O13	94.09 (10)
C5—C6—H6	120.2	O16—Ni3—N5	175.41 (12)
C7—C6—H6	120.2	O13—Ni3—N5	81.74 (11)
O3—C7—C8	118.1 (4)	O16—Ni3—O4	91.53 (10)
O3—C7—C6	123.8 (4)	O13—Ni3—O4	168.31 (11)
C8—C7—C6	118.1 (4)	N5—Ni3—O4	92.24 (11)
C7—C8—C9	120.1 (4)	O16—Ni3—N6	79.74 (12)
C7—C8—H8	119.9	O13—Ni3—N6	94.34 (12)
C9—C8—H8	119.9	N5—Ni3—N6	102.40 (12)
C4—C9—C8	122.2 (4)	O4—Ni3—N6	96.75 (11)
C4—C9—H9	118.9	O16—Ni3—O1M	80.37 (9)
C8—C9—H9	118.9	O13—Ni3—O1M	90.18 (11)
C7—O3—H3	109.5	N5—Ni3—O1M	97.66 (10)
C11—N2—Ni1	106.6 (2)	O4—Ni3—O1M	80.65 (10)
C11—N2—H2A	110.4	N6—Ni3—O1M	159.86 (10)
Ni1—N2—H2A	110.4	C38—N5—Ni3	112.1 (2)
C11—N2—H2B	110.4	C38—N5—H5A	109.2
Ni1—N2—H2B	110.4	Ni3—N5—H5A	109.2
H2A—N2—H2B	108.6	C38—N5—H5B	109.2
C10—O4—Ni1	113.5 (2)	Ni3—N5—H5B	109.2
C10—O4—Ni3	130.1 (2)	H5A—N5—H5B	107.9
Ni1—O4—Ni3	99.65 (10)	C37—O13—Ni3	114.5 (2)
O5—C10—O4	124.9 (4)	O13—C37—O14	122.0 (3)
O5—C10—C11	119.2 (3)	O13—C37—C38	120.7 (3)
O4—C10—C11	115.8 (3)	O14—C37—C38	117.3 (3)
N2—C11—C12	112.5 (3)	N5—C38—C39	112.5 (3)
N2—C11—C10	109.0 (3)	N5—C38—C37	110.4 (3)
C12—C11—C10	114.6 (3)	C39—C38—C37	114.1 (4)
N2—C11—H11	106.7	N5—C38—H38	106.4
C12—C11—H11	106.7	C39—C38—H38	106.4
C10—C11—H11	106.7	C37—C38—H38	106.4
C13—C12—C11	114.2 (3)	C40—C39—C38	113.7 (4)
C13—C12—H12A	108.7	C40—C39—H39A	108.8
C11—C12—H12A	108.7	C38—C39—H39A	108.8
C13—C12—H12B	108.7	C40—C39—H39B	108.8
C11—C12—H12B	108.7	C38—C39—H39B	108.8
H12A—C12—H12B	107.6	H39A—C39—H39B	107.7
C18—C13—C14	116.2 (4)	C41—C40—C45	115.2 (5)
C18—C13—C12	120.5 (4)	C41—C40—C39	122.0 (4)
C14—C13—C12	123.3 (4)	C45—C40—C39	122.8 (4)
C13—C14—C15	123.6 (4)	C42—C41—C40	123.1 (5)
C13—C14—H14	118.2	C42—C41—H41	118.5
C15—C14—H14	118.2	C40—C41—H41	118.5
C14—C15—C16	118.2 (4)	C41—C42—C43	120.6 (5)
C14—C15—H15	120.9	C41—C42—H42	119.7
C16—C15—H15	120.9	C43—C42—H42	119.7
O6—C16—C17	123.5 (4)	O15—C43—C42	123.4 (4)
O6—C16—C15	117.0 (4)	O15—C43—C44	116.7 (4)
C17—C16—C15	119.4 (4)	C42—C43—C44	119.9 (4)
C16—C17—C18	120.2 (4)	C43—C44—C45	119.1 (4)
C16—C17—H17	119.9	C43—C44—H44	120.5
C18—C17—H17	119.9	C45—C44—H44	120.5
C13—C18—C17	122.2 (4)	C44—C45—C40	122.1 (4)
C13—C18—H18	118.9	C44—C45—H45	118.9
C17—C18—H18	118.9	C40—C45—H45	118.9
C16—O6—H6A	109.5	C43—O15—H15A	109.5
O7—Ni2—O16	170.42 (10)	C47—N6—Ni3	110.1 (2)
O7—Ni2—O10	94.22 (10)	C47—N6—H6B	109.6
O16—Ni2—O10	92.13 (11)	Ni3—N6—H6B	109.6
O7—Ni2—O1M	93.04 (10)	C47—N6—H6C	109.6
O16—Ni2—O1M	80.85 (9)	Ni3—N6—H6C	109.6
O10—Ni2—O1M	81.11 (9)	H6B—N6—H6C	108.2
O7—Ni2—N3	82.47 (11)	C46—O16—Ni3	118.9 (2)
O16—Ni2—N3	90.61 (12)	C46—O16—Ni2	139.0 (2)
O10—Ni2—N3	174.38 (12)	Ni3—O16—Ni2	101.34 (11)
O1M—Ni2—N3	94.50 (11)	O17—C46—O16	122.8 (4)
O7—Ni2—N4	93.59 (11)	O17—C46—C47	120.9 (4)
O16—Ni2—N4	94.65 (11)	O16—C46—C47	116.3 (3)
O10—Ni2—N4	79.10 (10)	C46—C47—N6	111.6 (3)
O1M—Ni2—N4	159.52 (10)	C46—C47—C48	108.9 (3)
N3—Ni2—N4	105.56 (11)	N6—C47—C48	110.6 (3)
C20—N3—Ni2	110.8 (2)	C46—C47—H47	108.6
C20—N3—H3C	109.5	N6—C47—H47	108.6
Ni2—N3—H3C	109.5	C48—C47—H47	108.6
C20—N3—H3D	109.5	C49—C48—C47	114.6 (3)
Ni2—N3—H3D	109.5	C49—C48—H48A	108.6
H3C—N3—H3D	108.1	C47—C48—H48A	108.6
C19—O7—Ni2	115.0 (3)	C49—C48—H48B	108.6
O8—C19—O7	121.6 (4)	C47—C48—H48B	108.6
O8—C19—C20	118.1 (3)	H48A—C48—H48B	107.6
O7—C19—C20	120.3 (4)	C54—C49—C50	116.2 (4)
N3—C20—C19	110.3 (3)	C54—C49—C48	122.4 (4)
N3—C20—C21	111.5 (3)	C50—C49—C48	121.5 (4)
C19—C20—C21	111.0 (3)	C51—C50—C49	121.6 (4)
N3—C20—H20	108.0	C51—C50—H50	119.2
C19—C20—H20	108.0	C49—C50—H50	119.2
C21—C20—H20	108.0	C50—C51—C52	121.5 (4)
C22—C21—C20	113.8 (3)	C50—C51—H51	119.3
C22—C21—H21A	108.8	C52—C51—H51	119.3
C20—C21—H21A	108.8	O18—C52—C53	117.4 (4)
C22—C21—H21B	108.8	O18—C52—C51	124.8 (4)
C20—C21—H21B	108.8	C53—C52—C51	117.8 (4)
H21A—C21—H21B	107.7	C54—C53—C52	121.1 (4)
C23—C22—C27	117.2 (3)	C54—C53—H53	119.5
C23—C22—C21	121.2 (4)	C52—C53—H53	119.5
C27—C22—C21	121.4 (3)	C53—C54—C49	121.8 (4)
C22—C23—C24	122.6 (4)	C53—C54—H54	119.1
C22—C23—H23	118.7	C49—C54—H54	119.1
C24—C23—H23	118.7	C52—O18—H18A	109.5
C25—C24—C23	118.5 (4)	O1M—C1M—H1M1	109.5
C25—C24—H24	120.7	O1M—C1M—H1M2	109.5
C23—C24—H24	120.7	H1M1—C1M—H1M2	109.5
O9—C25—C26	119.0 (4)	O1M—C1M—H1M3	109.5
O9—C25—C24	121.2 (3)	H1M1—C1M—H1M3	109.5
C26—C25—C24	119.8 (4)	H1M2—C1M—H1M3	109.5
C25—C26—C27	120.0 (4)		

### Hydrogen-bond geometry (Å, º)

**Table:**

*D*—H···*A*	*D*—H	H···*A*	*D*···*A*	*D*—H···*A*
N1—H1*A*···O15^i^	0.90	2.26	3.082 (4)	153
N1—H1*B*···O18^i^	0.90	2.20	3.042 (4)	157
O3—H3···O3*M*	0.82	1.79	2.601 (5)	170
O6—H6*A*···O14^ii^	0.82	1.88	2.685 (4)	166
N3—H3*C*···O13	0.90	2.36	3.174 (4)	150
O9—H9*A*···O2^iii^	0.82	1.78	2.597 (4)	173
N4—H4*B*···O17	0.90	2.29	3.050 (5)	142
O12—H12···O2*M*^iii^	0.82	1.90	2.669 (7)	155
N5—H5*A*···O1	0.90	2.46	3.251 (4)	146
N5—H5*A*···O9^iv^	0.90	2.54	3.216 (4)	133
O15—H15*A*···O8^v^	0.82	2.02	2.815 (4)	164
O15—H15*A*···O7^v^	0.82	2.47	2.983 (4)	122
O18—H18*A*···O11^v^	0.82	1.84	2.638 (4)	163
C1*M*—H1*M*1···O1	0.96	2.52	3.042 (4)	114
C30—H30*A*···O11	0.97	2.55	2.893 (5)	101
C38—H38···O9^iv^	0.98	2.38	3.178 (5)	138
C54—H54···O5	0.93	2.42	3.338 (5)	167

Symmetry codes: (i) *x*, *y*, *z*+1; (ii) −*x*+1, *y*−1/2, −*z*+1; (iii) *x*−1, *y*, *z*; (iv) *x*+1, *y*, *z*; (v) *x*, *y*, *z*−1.
